# Supplementary material for: Immune Response Modulation by Pseudomonas aeruginosa Persister Cells
Source: mBio. 2023 Mar 15;14(2):e00056-23. doi: 10.1128/mbio.00056-23 (PMC10128020; doi:10.1128/mbio.00056-23)
Supplement: FIG S1 [file mbio.00056-23-s0001.pdf]

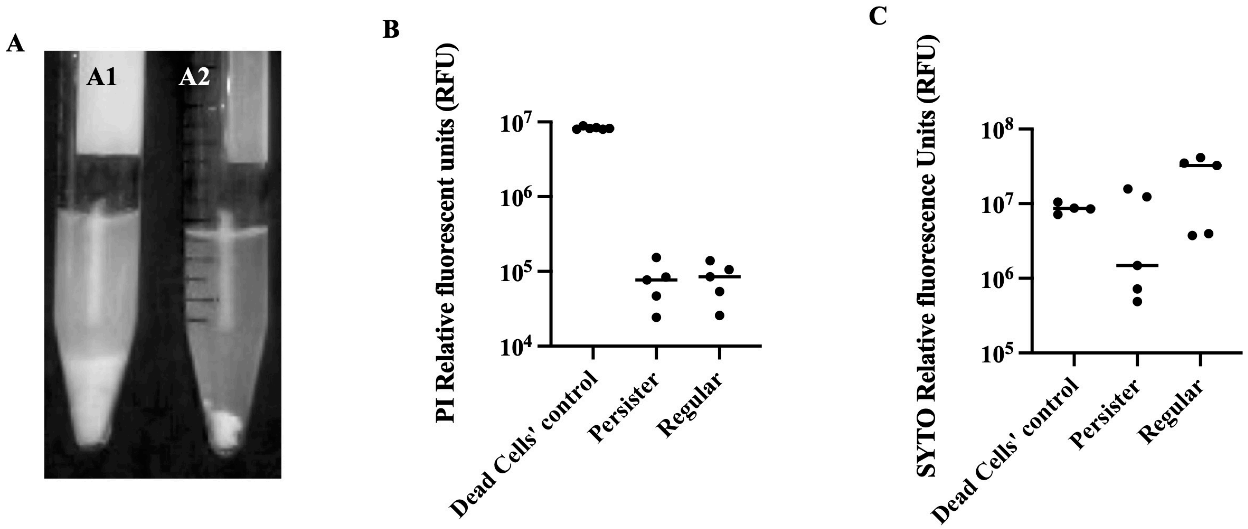

Figure S1. Cells remaining following selection. Regular vegetative cells and persister cells of *P. aeruginosa* PA14 were isolated from stationary phase planktonic cultures. Cells exposed to saline were named regular vegetative cells (A1) and cells exposed to ciprofloxacin (20 mg/L) in saline were named persister cells (A2). Subsequently, isolated/selected cells were stained with SYTO9 and propidium iodide and the % of dead cells was calculated (B).
