# Supplementary material for: Immune Response Modulation by Pseudomonas aeruginosa Persister Cells
Source: mBio. 2023 Mar 15;14(2):e00056-23. doi: 10.1128/mbio.00056-23 (PMC10128020; doi:10.1128/mbio.00056-23)
Supplement: FIG S2 [file mbio.00056-23-s0002.pdf]

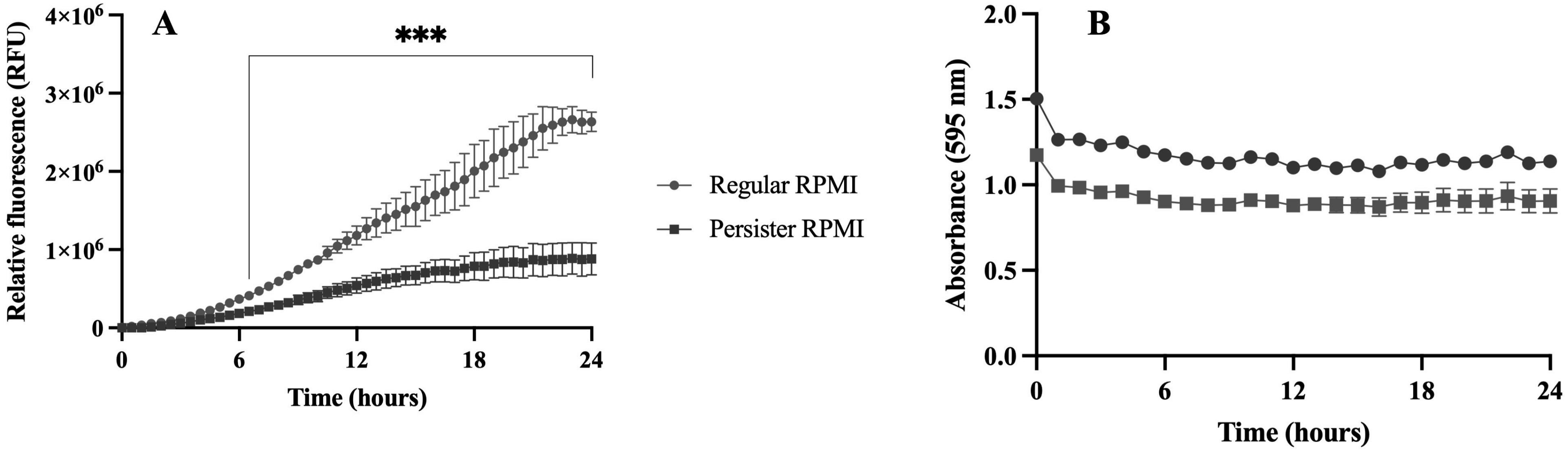

Figure S2. Bacterial growth and metabolism of regular vegetative and persister cell. Bacterial populations of *P. aeruginosa* PA14 and MPAO1 attTn7::P(A1/04/03)::GFPmut were isolated and resuspended in RPMI medium. Constitutive fluorescence of MPAO1 attTn7::P(A1/04/03)::GFPmut (A) and absorbance of PA14 (B) were monitored for 24 hours. Results were analyzed according T-test (***P<0.001) and are presented as mean ± SD.
