# Supplementary material for: Immune Response Modulation by Pseudomonas aeruginosa Persister Cells
Source: mBio. 2023 Mar 15;14(2):e00056-23. doi: 10.1128/mbio.00056-23 (PMC10128020; doi:10.1128/mbio.00056-23)
Supplement: FIG S3 [file mbio.00056-23-s0003.pdf]

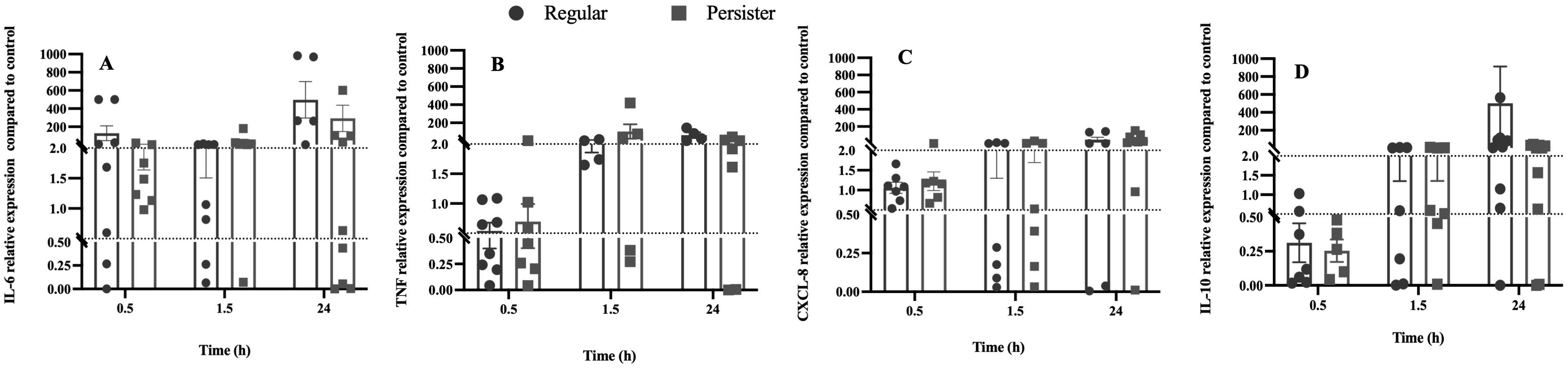

Figure S3. Macrophage cytokine gene expression by macrophages. The relative gene expression of 4 cytokines IL-6 (A), TNF (B), CXCL-8 (C), and IL-10 (D) was quantified for: 0.5, 1.5, and 24 h of infection (MOI of 10) with *P. aeruginosa* regular and persister cells. The relative expression level for *P. aeruginosa* persister and regular cells was compared to un-infected controls. Results shown consist of at least 4 experiments. The CT value of the housekeeping gene *gadph* remained constant throughout the different treatments (P>0.5 by ANOVA and no difference between treatments by Tukey's multiple-comparison test). A significant change was considered to occur when a 2-fold change in the relative expression level occurred.
